# Arrhythmia Caused by a *Drosophila* Tropomyosin Mutation Is Revealed Using a Novel Optical Coherence Tomography Instrument

**DOI:** 10.1371/journal.pone.0014348

**Published:** 2010-12-17

**Authors:** Lisha Ma, Adrian Bradu, Adrian Gh. Podoleanu, James W. Bloor

**Affiliations:** 1 Cell and Developmental Biology Group, School of Biosciences, University of Kent, Canterbury, Kent, United Kingdom; 2 Applied Optics Group, School of Physical Sciences, University of Kent, Canterbury, Kent, United Kingdom; Tufts University, United States of America

## Abstract

**Background:**

Dilated cardiomyopathy (DCM) is a severe cardiac condition that causes high mortality. Many genes have been confirmed to be involved in this disease. An ideal system with which to uncover disease mechanisms would be one that can measure the changes in a wide range of cardiac activities associated with mutations in specific, diversely functional cardiac genes. Such a system needs a genetically manipulable model organism that allows *in vivo* measurement of cardiac phenotypes and a detecting instrument capable of recording multiple phenotype parameters.

**Methodology and Principal Findings:**

With a simple heart, a transparent body surface at larval stages and available genetic tools we chose *Drosophila melanogaster* as our model organism and developed for it a dual *en-face*/Doppler optical coherence tomography (OCT) instrument capable of recording multiple aspects of heart activity, including heart contraction cycle dynamics, ostia dynamics, heartbeat rate and rhythm, speed of heart wall movement and light reflectivity of cardiomyocytes *in situ*. We applied this OCT instrument to a model of Tropomyosin-associated DCM established in adult *Drosophila*. We show that DCM pre-exists in the larval stage and is accompanied by an arrhythmia previously unidentified in this model. We also detect reduced mobility and light reflectivity of cardiomyocytes in mutants.

**Conclusion:**

These results demonstrate the capability of our OCT instrument to characterize in detail cardiac activity in genetic models for heart disease in *Drosophila*.

## Introduction

Dilated cardiomyopathy (DCM) is a progressive pathological cardiac condition characterized by an enlarged heart with impaired contractility, defects that often lead to heart failure. To date a wide range of genes, from those involved in mechanobiochemical signaling to components of the contractile architecture, have been confirmed to cause DCM when defective [1]–[4]. This suggests that a large molecular genetic network regulates the development of this disease. Therefore, in order to shed light on disease mechanisms, evaluation of each genes cardiac impact is necessary. With this ultimate goal in mind, one method allowing us to move forward is to use a genetically tractable model organism to investigate cardiac function of individual genes. Such an approach requires a model organism with two properties, a methodology that allows individual genes to be knocked-down specifically in the heart, and a heart that is easily accessible to imaging. In addition, an imaging system is required that can accurately and easily measure subtle changes in multiple parameters of cardiac function.

The fruitfly, *Drosophila melanogaster*, is an ideal model for this experimental approach. It has an open circulatory system, a cardiovascular organ called the dorsal vessel driving hemolymph flow around the body [5], [6]. The dorsal vessel is a simple tube formed by a single layer of cardiomyocytes. It is divided into two morphologically distinct functional domains: the thin anterior aorta serving as an outflow tract and the broad posterior heart acting as a rhythmic pump. It resides next to the body surface so that it allows easy observation *in vivo*. Despite the obvious morphological differences between the *Drosophila* and vertebrate hearts there is a remarkable degree of conservation between the two, not only in the characteristic rhythmic cardiomyocyte contraction, but also in the genetic networks that regulate early heart development [7]–[13] and aspects of heart physiology [14], [15]. Indeed in both *Drosophila* and vertebrate hearts mutations in homologous genes cause DCM-like phenotypes [16], [17]. Moreover, genetic tools available in *Drosophila* provide the ability to knockdown more than 90% of the genome using RNAi specifically targeted to the dorsal vessel [17].

Key to the potential of this *Drosophila*-based heart model is an imaging technique that can accurately record cardiac dynamics. Several approaches have been reported. In the embryo cardiac dynamics can be recorded *in situ* using fluorescence microscopy combined with cardiac specific expression of GFP [18]. This has the potential to identify genes involved in the development and initial activity of the dorsal vessel, but as contractile activity only starts an hour before hatching and in larvae the dorsal vessel becomes partially obscured by the overlying fat body, this technique is limited in its ability to detect genes involved in progressive heart disease. An alternative and powerful technique pioneered in adult cardiac imaging, utilizes a digital video camera with differential interference contrast optics to image the edge of the heart wall [19]. However, pigment in the adult cuticle limits light penetration, thus imaging has to be performed in dissected animals. This technique has recently been applied to whole *Drosophila* larvae [20], however issues with the obscuring fat body remain. The final approach is to use optical coherence tomography (OCT) [21]. This is a real time label-less imaging technique with mm range of depth penetration that generates an image by interfering a reference light with light back-scattered from the specimen. Tissues and cells are distinguished from surrounding tissue fluid due to their different light reflectivity [22]. OCT has been applied to the study of cardiac dynamics in intact adult *Drosophila* [16], [23], as well as in mouse [24] and avian embryos [25]. However, real time recording using OCT has been limited to 2-dimensional (lateral × depth) cross sectional views of the heart, thus sampling contractile activity at a single point rather than along the full length of the organ. An OCT instrument that could image a plane along the longitudinal axis of the heart, producing an *en-face* image oriented as in conventional microscopy, could provide further functional information.

Here we describe an OCT instrument which can operate in two regimes. In the imaging regime, the system can acquire OCT images, with either cross sectional or *en-face* orientation, of the *Drosophila* larval heart. In the Doppler regime, movement of the heart wall is measured. We demonstrate the utility of this instrument in recording larval heart dynamics by applying it to the analysis of a mutation in the *Drosophila* muscle specific Tropomyosin gene previously shown to cause a DCM phenotype in the adult fly heart [16]. We show that a similar phenotype pre-exists at larval stage and demonstrate that this Tropomyosin defect also causes an arrhythmia characterized by increased length and frequency of heart pausing. Moreover, the OCT instrument uniquely enables assessment of cellular mobility and light reflectivity *in situ*. In combination with the genetic tools available in *Drosophila*, this instrument provides an excellent platform for future detailed study of the molecular mechanisms of DCM and arrhythmia.

## Results

### *En-face* imaging of wild type larval heart

Initially we applied *en-face* OCT (details are described in Materials and Methods section) to heart imaging of wandering stage 3^rd^ instar *w^1118^* control larvae. Previous studies have shown that the fly heart contracts in peristaltic waves that propagate along the heart [5], [18], [26], but there is discrepancy regarding the directionality of hemolymph flow. Slama and Farkas [26] reported that the larval heart beat is always uni-directional from the posterior towards the anterior, while Rizki [5] reported occasional reversals. This reversal contraction has also been observed in semi-dissected larval heart [27]. In all the *w^1118^* larvae (n = 60) imaged in this work, the dominant form of contraction was a peristaltic wave of caudal origin (see Movie S1). In 4 larvae (∼7%) peristaltic contractions were occasionally interspersed by short periods of twitching. In 13 larvae (∼22%) peristaltic contractions were interspersed with periods when the heart chamber shortened along its anterior-posterior axis, these longitudinal contractions appearing to initiate from the anterior.

**Figure 1:**
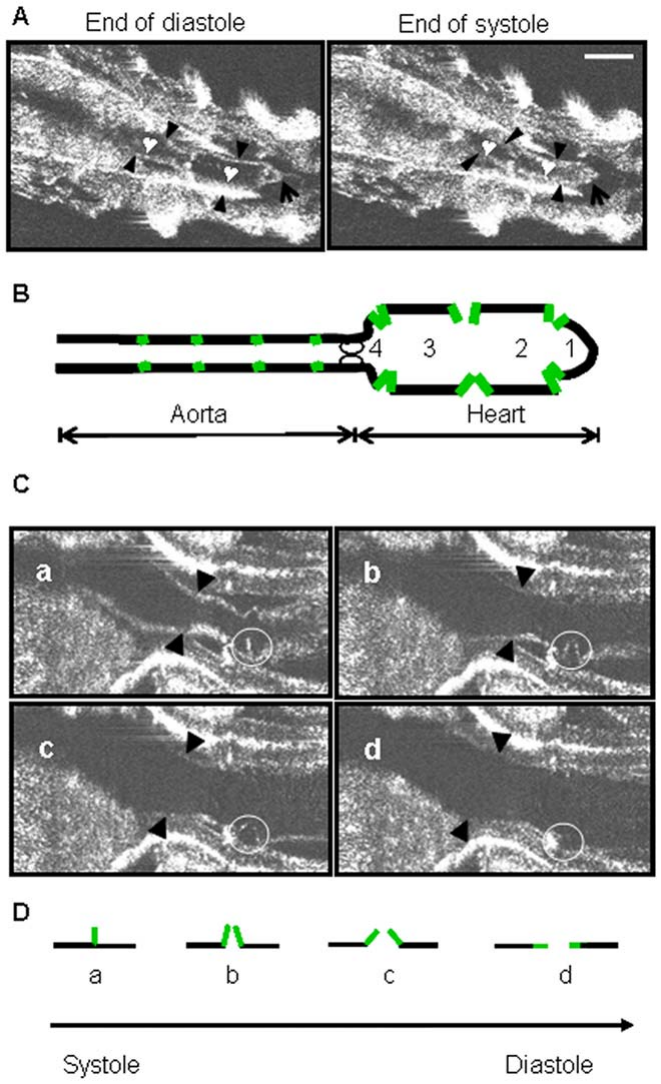
*Drosophila* 3^rd^ instar larval heart imaged using *en-face* OCT. **A**: *en-face* OCT images of the normal larval heart at the end of diastole and the end of systole. Arrow heads indicate the heart wall; the arrows indicate the caudal heart chamber; the heart signs indicate chambers 2 and 3 that are divided by a pair of protruding ostia structure. Bar size is 180 µm. **B**: Schematic drawing of the dorsal vessel (heart and aorta) modified from description by others [5], [29]. Posterior of larvae is oriented to the right throughout this report. Heart valve is shown as oval shape circles. Ostia cells (green) divide the heart wall (black) into 4 chambers. **C**: The cell pair of a single ostium (in white circle), showing their positions relative to the heart wall through a contraction cycle (a–d). Arrow heads indicate the heart wall. Bar size is 90 µm. **D**: Schematic illustration of the ostium through the systole/diastole cycle (from a, b, c to d) seen in C. The green lines represent the pair of ostium cells, and black lines represent the heart wall.

The larval heart chamber possesses three pairs of laterally located ostia along its axis [28], [29], and they divide the heart chamber into four sub chambers [5], as indicated in Fig. 1A, 1B. These ostia are heart gates, each of them a pair of specialized cells that serve as valves to regulate the inward flow of hemolymph into the heart [5], [6], [28]. Ostia dynamics have been described in the embryonic heart [18], the valves opening in a coordinated fashion as the heart relaxes, drawing hemolymph in, and closing at systole to push hemolymph forward into the aorta. Using our *en-face* OCT system similar dynamics in the larval heart were imaged (Fig. 1C, 1D).

### *En-face* imaging of the heart in Tropomyosin mutant larvae

**Figure 2:**
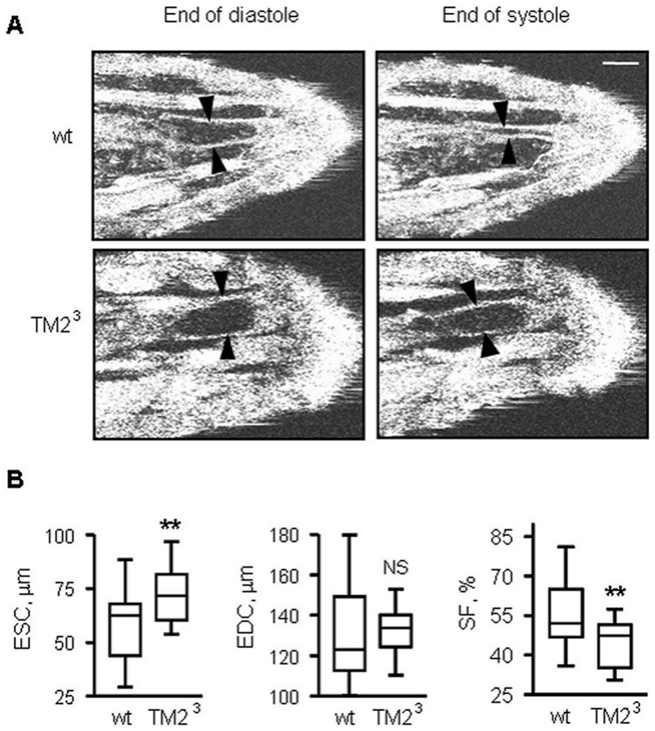
Dilated cardiomyopathy in Tropomyosin mutant larvae examined with *en-face* OCT. **A**: representative OCT images of the end of systole and the end of diastole in control (*w^1118^*) and Tropomyosin mutant (*TM2^3^*) larvae. Bar size is 180 µm. **B**: comparison of heart diameter parameters and shortening fraction between control and *TM2^3^* larvae. Heart size: Mean ± SEM in *w^1118^* (n = 21) and *TM2^3^* (n = 18). ESC: end of systolic caliber; EDC: end of diastolic caliber; SF  =  [(EDC - ESC)/EDC] ×100%. Data between control and *TM2^3^* was analyzed using the student t test. **: p<0.01, NS: p>0.05.

Adult *Drosophila* homozygous for the Tropomyosin II null mutant *TM2^3^* exhibit a DCM-like heart [16]. Using our OCT instrument we examined whether this mutation causes a heart defect at an earlier developmental stage. Compared to control *w^1118^* larvae, *TM2^3^* mutants showed a decreased shortening fraction (SF) (Fig. 2), indicating reduced cardiac contractility. In adult *TM2^3^* mutants, the dilated heart chamber results from enlargement at both maximal systole and maximal diastole [16]. At the earlier larval stage, heart chamber dilation is mainly due to failure of the heart to fully contract, the enlargement of chamber diameter being statistically significant only at maximal systole. This demonstrates that the tropomyosin mutation causes a DCM-like phenotype as early as the larval stage. Interestingly the degree of the dilation defect is consistent between larval and adult stages [16], in both cases the shortening fraction being decreased by 19% of the control value.

In addition, although the same proportion of *w^1118^* and *TM2^3^* larvae exhibited chamber shortening contractions (Table 1), there was a noticeable increase in the frequency at which these contractions occurred in the tropomyosin mutant larvae. An example is shown in movie S2 (see Supplement Information). The cause of this phenomenon is not clear.

**Table 1:** Heart rate analyzed using the OCT imaging and audio recording data.

	Co`ntrol (*w^1118^*), %	*TM2^3^*, %
**Imaging analysis**	n* = 38	n = 47
Wavy contraction	64	62
Occasional chamber shortening	28	−
Dominant chamber shortening	−	27
Chamber shortening only	0	4
Pauses	8	34
**Audio recording analysis**	n = 27	n = 27
Regular rate	82	63
Irregular rate	11	11
Pause (>2 sec)	7	26

*: number of animal used.

### Heart rate of control and Tropomyosin mutant larvae

**Figure 3:**
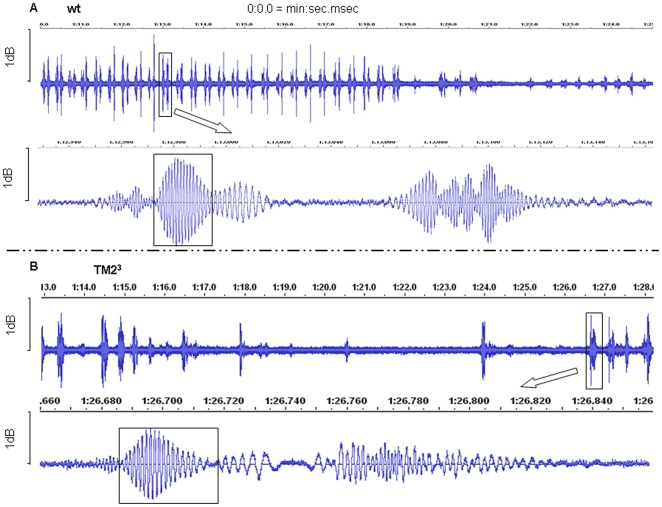
Larval heart rate and arrhythmia in Tropomyosin mutant. **A**: a typical control heart audio tracing. **B**: a representative Tropomyosin mutant heart audio tracing. Boxed double spikes of the tracing in top panels of both A and B are one heart beat; both bottom panels are the enlarged trace of each of the indicated heart beat in the top panels. The higher frequencies indicated in the boxed part in each heart tracing (both bottom panels) were used in calculating the velocity of Doppler signals.

**Figure 4:**
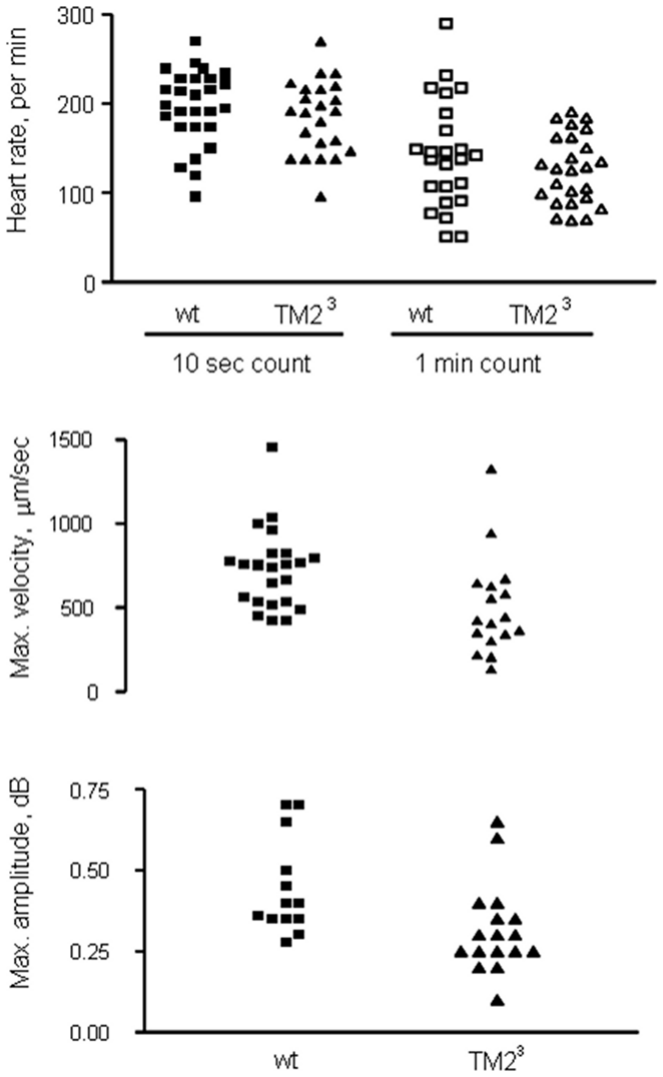
Summarised heart rate, velocity and light reflectivity of cardiomyocyte deduced from Doppler OCT-based audio recording. The following information was generated from the Doppler audio recording. Top panel is heart rate in 1 minute. In 10 sec heart rate counting group, Mean ± SEM for controls is 154±12.09 (n = 25), for *TM2^3^* is 129±10.4 (n = 15). In 1 min heart rate counting group, Mean ± SEM for controls is 196±8 (n = 27), for *TM2^3^* is 185±10 (n = 12), p>0.05 in both counting groups. Middle panel is the max. velocity of Doppler signals, representing cardiomyocyte's mobility. Mean ± SEM (µm/sec) for control group is 726±240 (n = 23), for *TM2^3^* group is 502±293, p = 0.012. Bottom panel is the amplitude, representing cardiomyocyte's light reflectivity, of Doppler signals. Mean ± SEM (dB) for the control group is 0.44±0.04 (n = 13) and for the *TM2^3^* 0.32±0.03 (n = 17), p = 0.0214.

It is well documented that the fruitfly heartbeat is irregular. It has been noticed previously that short periods of heart pausing are associated with eating activity and with preparation for crawling in *Drosophila* larvae [5]. We noticed a similar association of body movement with heart pausing in our *en-face* imaging observations. This slowed heart rate prior to and post body crawling contraction was confirmed (an example of *w^1118^*: Audio Recording S1) by our Doppler recordings (as described in Materials and Methods). Typical recordings of control and *TM2^3^* mutant hearts are shown in Fig. 3. To take this kind of pausing into consideration, heart beat in both control and *TM2^3^* mutant larvae was analyzed by two methods, counting the number of heart beats over either periods of 10 seconds or 1 minute. With the 10 second period, average heart rates of mutants and controls were similar, but using the 1 minute period a drop of 14% in heart rate was seen in the *TM2^3^* mutants although the difference was not statistically significant (Fig. 4). Note that the heart rate determined using the 1 minute period was lower than that calculated using the 10 second period in both groups. These data suggest that the dilated hearts of *TM2^3^* mutants can have similar pumping capacity as wild type over short time periods, but over longer periods there is a trend towards reduced pumping capacity. Further studies are needed to confirm this finding.

### Arrhythmia is increased in the heart of Tropomyosin mutant larvae

Heart arrhythmia is associated with DCM-causing tropomyosin mutations in humans, but has not been apparent in the adult *Drosophila TM2^3^* mutant. Taking into consideration the natural irregularity of fly heartbeat, we therefore screened Doppler audio recordings for larval heart pausing of longer than 2 seconds. Doppler data was cross-examined with *en-face* OCT imaging from the same animal to confirm the occurrence of contractile pauses. The Doppler data showed that, compared to control larvae where pausing was a relatively rare occurrence (seen in ∼7% of larvae, n = 27), heart pausing increased to ∼26% (n = 27) in *TM2^3^* mutants (Table 1). An example is shown in Movie S3 and Fig. 3. In addition, pauses were more frequent and lasted longer, up to 30 seconds in mutants compared to less than 10 seconds maximum in control animals.

### Reduced cellular mobility and light reflectivity in the heart of Tropomyosin mutant larvae

As shown in the bottom panels of Fig. 3A and 3B, the Doppler frequency was not constant throughout one heartbeat cycle. According to the Doppler shift principle, a higher frequency is produced when the distance between the detecting point and the moving heart wall diminishes and a lower frequency is produced when the distance increases. Therefore, a higher frequency is recorded when the heart wall moves toward the instrument. In order to compare the velocities of heart wall motion between *TM2^3^* mutants and control larvae, only the part of the tracing showing the highest frequency within a heartbeat (boxed in Fig. 3A, 3B, bottom panels) was used in calculating the velocity (as described in Materials and Methods). Results showed that the velocity of movement of the heart chamber wall decreased by 31% in *TM2^3^* mutants compared to controls (Fig. 4).

Amplitude recorded by the Audicity plot (Fig. 3) represents the intensity of the Doppler signal and is a measure of the optical density of the tissue, in this case the heart wall. The cardiomyocytes that make up the heart wall are densely packed with myofibrils, the highly developed ultrastructural units of contraction. Thus, signal amplitude can be taken as a rough measure of the integrity of the myofibril ultrastructure. Analysis of the maximum amplitude of the Doppler signal observed in each animal shows a significant reduction in the *TM2^3^* mutant heart (Fig. 4), indicative of a reduced ultrastructural density in this mutant cardiomyocyte.

## Discussion

In order to improve the sensitivity of the methodologies used to dissect the genetic networks underlying dilated cardiomyopathy, we built a dual-regime OCT instrument dedicated for the *in situ* measurement of multiple cardiac parameters in the *Drosophila* larval heart. Measurements from this instrument allow the organ level phenotype to be assessed, including heart size, mode of heart contraction, ostia dynamics, heart rate and heart rhythm. In addition, the Doppler regime uniquely provides measurements for assessing the mobility of the cardiomyocyte *in situ*. The light reflectivity of the cardiomyocyte, as indicated by the amplitude of the Doppler signal, is another unique parameter provided by this detecting instrument. Application of our instrument to larvae homozygous for a Tropomyosin mutant known to cause DCM in the adult fly reveals for the first time, to our knowledge, that an arrhythmia is associated with this mutation in this organism. Both mobility and light reflectivity of cardiomyocytes were also found to be affected in this mutant. In addition recording of ostia dynamics provides an avenue for studying the impact of hemolymph dynamics on cardiac physiology and on fly heart development. Such a role for fluid dynamics has been demonstrated in early chick heart development [25]. These results demonstrate that application of our OCT instrument in this model organism enables detailed characterization of cardiac gene activities across multiple hierarchical structures *in situ*. In comparison, a recent *Drosophila* genome wide screen identified a number of candidate cardiac genes by screening for adult lethality associated with a combination of RNAi knockdown and cardiac stress [17]. Surprisingly, muscle specific Tropomyosin was not identified by this screen despite its known adult cardiac phenotype. Thus, while more time consuming, a screening approach in which detailed characterization of cardiac function is performed using our OCT system is likely to be much more comprehensive in identifying potential cardiac disease genes.

The reduced light reflectivity in the *TM2^3^* mutant found in this work suggests a reduced density of cellular ultrastructure content. Supporting evidence for this suggestion comes from the observation that a mutant in Tropomyosin's partner protein, Troponin I exhibits disorganized cardiac ultrastructure [16]. Furthermore, a massive reduction of Doppler signal amplitude is associated with substantial loss of myofibrils in larval hearts depleted of the β_PS_ integrin subunit (our unpublished data).

Our data reaffirms that, predominantly, heart contraction takes the form of a peristaltic wave of posterior origin. In embryos [18] and larvae (this work), these contractile waves are coordinated with ostia dynamics and the opening of the aortic valve to generate an anterior-ward flow of hemolymph through the aorta. In the normal heart, these peristaltic heart beats are interspersed with short periods of either shortening contractions, where the heart chamber shortens along its anterior-posterior length, or fast twitching. Because the twitching contractions appeared to be coordinated or synchronized in the whole heart, they could be caused by interference between the caudal cardiac pacemaker and neural activity from the anterior aorta directing retrograde contractions. Such interference has been suggested in the adult heart where retrograde contractions are directed by neural connections to the anterior aorta. In this scenario, the shortening contractions we observed would be larval retrograde contractions described by some observers [5], [18], but not others [26], where anterior neural activity has overcome the cardiac pacemaker. Such a scenario is supported by the fact that fast twitch contractions and shortening contractions appear to be mutually exclusive and are not observed in the same larvae over the period of observation. If this hypothesis is true it is interesting to note that in the Tropomyosin model of DCM we observed larvae in which shortening contractions predominated suggesting that contractions initiated by the cardiac pacemakers are preferentially affected in this mutant.

Arrhythmias occur in one third of the patients suffering from DCM associated with mutation in the α-tropomyosin gene [30]. An altered sensitivity of myofilaments to calcium ion concentration has been observed in an animal model with a mutation of this gene [31]. Arrhythmia associated with defects in ion channels have been reported in *Drosophila* [29], [32]. Abnormal irregular heart beat can also be induced by drugs, different ionic solutions and by physical injury of the heart in the semi-dissected fly [33]. Investigating the mechanisms underlying these affects in combination with electrophysiology studies, is a further potential application of our dual OCT imaging and Doppler instrument. In particular, in injured cells combining monitoring of structure damage with OCT imaging with measurement of electrophysiology and contractile functionality may identify similarities between the model injury and infarct in heart-attack patients. Moreover, a comparative study of arrhythmias caused by functionally diverse genes is expected to generate a better understanding of the causative mechanism. The phenotypic resemblance between *Drosophila* and human in tropomyosin-associated arrhythmia in DCM suggests a conserved disease mechanism between species. Thus our OCT instrument provides a powerful tool for further investigation of the molecular mechanisms of the DCM coupled arrhythmia.

## Materials and Methods

### *En-face* OCT imaging and Doppler instrument dedicated for *in situ* analysis of cardiac function in *Drosophila* larvae

The ideal instrument for recording cardiac dynamics in *Drosophila* requires the following attributes. It must be capable of imaging the heart in the intact animal, to do so it must be capable of penetrating the overlying cuticle and be capable of imaging at depth. Ideally orthogonal images of the heart should be acquired so that the full length of the organ can be observed. In addition, as the *Drosophila* larval heart beats between 3.5 and 4 times a second [26], the rate of image capture must exceed this frequency.

OCT has several of these attributes. It is a label-less imaging technique that generates an image from interference between an internal reference beam and light reflected by the target. It is capable of imaging at depth through the cuticle.

Previous studies that applied OCT to imaging of the adult *Drosophila* heart have produced cross-sectional images to depict the dynamics of heart diameter, and therefore heart rate, in real time [16], [23]. However, the specific technique used in these studies, spectral domain OCT, cannot generate *en-face* oriented images of the *Drosophila* heart at the required frame rate, (our initial studies on a SD-OCT system operating at 29 kHz rate still, in spite of its high line rate, required 5 seconds to acquire the whole OCT data volume [34]). To resolve this issue we turned to *en-face* time domain (TD)-OCT that can, in principle, achieve high speeds of frame acquisition by using resonant scanners [35]. Another reason for opting for a TD-OCT method is due to its compatibility to dynamic focus, the ability to move both the coherence gate and the focus gate together. This makes the TD-OCT method better suited to microscopy applications. Using a resonant scanner at 2 kHz, our TD-OCT imaging system can produce *en-face* images at up to 5 Hz rate, slightly faster than mean heart rate in *Drosophila* larvae.

Although the 5 Hz acquisition rate has largely improved our capability of following the heart rate, we are still missing important parts of the heart wall motion during each beat. To address this deficiency, we complemented the information acquired during the imaging process with that acquired during Doppler signal recording. In this regime, the heart wall motion is recorded in real time. This technique exploits the fact that a moving light-scattering object imposes a Doppler shift to the frequency of the scattered light that is proportional to the velocity of the movement of the object. In our instrument a Doppler shift was produced when the distance between fixed detecting focal point and heart wall changes due to the contraction/relaxation movement of the latter. The wavelength of the SLD light source, together with the speed of movement of the heart wall, place the Doppler frequency within the audio range; hence our instrument performs like a stethoscope and the Doppler signal may be plotted as a sound signal using Audicity software. A typical heartbeat audio recording from a control larva is shown in the supplement material (Audio Recording S1), and the plot is shown in Fig. 3A. Closely neighboring double peaks of high-density tracings (Fig. 3A, top panel) represent a single heartbeat.

**Figure 5:**
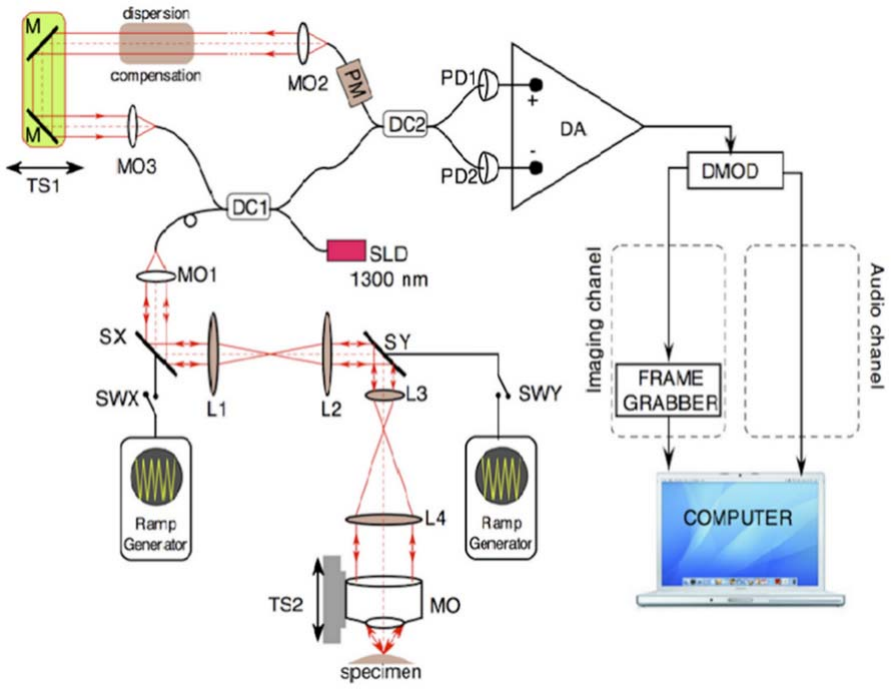
Diagram of the dual *en-face*/Doppler OCT. **SLD**: super-luminescent diode, **SX**: resonant scanning mirror, **SY**: galvanometer scanning mirror, **SWX/Y**: electrical switchers used to stop/start the scanners, **DC1,2**: directional couplers, **MO1-4**: microscope objectives, **M**: flat mirrors, **PD1,2**: photo-detectors, **PM**: polarization controller, **TS1/2**: computer controlled translation stages, **DMOD**: demodulator, **DA**: differential amplifier.

A schematic diagram of the dual *en-face* imaging/Doppler signal recording OCT system is shown in Figure 5. The technical details of the system are as follows. A pigtailed super-luminescent diode (SLD, SuperLum, Moscow) emitting at 1300 nm and having a spectral bandwidth of 65 nm is used which determines an OCT longitudinal resolution of around 17.3 µm in the sample. Light from the SLD source is injected into the system via a first directional coupler (DC1) that splits the light towards the probing and the reference arm of the interferometer. The probing beam is sent via the galvanometer scanners SX and SY to the specimen (*Drosophila* larval heart). Two telescopes conveniently alter the diameter of the beam in order to match the aperture of different elements in the probing path and convey a probing beam of around 8 mm in diameter through the microscope objective MO's pupil plane. The two transverse scanners, SX and SY, are separated here using a telescope in order to project a flat wave-front on the target under high numerical aperture (NA). Lenses L1, L2 and L4 have a focal length of 7.5 cm, while lens L3 has a focal length of 3 cm. The MO is a scan lens (focal length 1.8 cm) specially designed by ThorLabs to prevent image degradation and distortion during scanning. Hence, a lateral resolution better than 4.3 µm in the *en-face* OCT images is obtained (determined by imaging a USAF test target). To maintain this lateral resolution throughout the whole depth of the specimen, tracking of coherence gate and focus position (dynamic focus) has been implemented by simultaneously adjusting the two arms of the interferometer via the computer driven translation stages TS1 and TS2.

Light back-scattered by the specimen passes a second time through the object arm, guided via the first directional coupler towards the second single mode directional coupler where it interferes with that coming from the reference arm. Both output fibers from the second coupler are connected to two pin photo-detectors, PD1 and PD2, in a balanced photo-detection unit constructed using a differential amplifier, DA. The OCT signal is rectified and low pass filtered in the demodulator DMOD. A computer-driven translation stage, TS1, is used to alter the reference path length to select different depths for C-scans while acquiring stacks of C-scans as well as scanning the depth in the B-scan acquisition mode. The scanning procedure is similar to that used in any confocal microscope, where the fast scanning is *en-face* (line rate, using the scanner SX) and the frame scanning is much slower (at the frame rate, using the scanner SY). The frame grabber in Figure 5 is controlled by TTL signals from the generators driving the SX-scanner and the SY-scanner. The SX resonant scanner is driven with a ramp at 2 kHz and the SY galvo-scanner with a ramp at 5 Hz. In this way, an *en-face* image, in the plane (x, y) is generated at constant depth. The next *en-face* image at a new depth is then generated by moving the translation stage, TS1, in the reference arm of the interferometer and repeating the (x, y) scan. *En-face* images with a size of 1.5 : 1 in width over height in this report are obtained using suitable amplitudes for the voltages applied to the X and Y-galvoscanners.

To switch the system to the audio (stethoscope) regime, the scanning of the beam across the specimen is interrupted using switches SWX and SWY. Hence, the recorded interferometric signal, low-pass filtered by the DMOD block, is exclusively due to projection of movement of the heart wall along the axial direction of the objective lens.

### *Drosophila* stocks, larval heart imaging and heartbeat audio recording

The *w^1118^* and Tropomyosin II mutant *TM2^3^* stocks were obtained from the Bloomington *Drosophila* Stock Centre (Bloomington, IN) and raised on standard fly food at 25°C. The *w^1118^* is control strain carrying a mutation in the *w* (*white*) gene which affects the production of red pigment in the adult eye but has no known effect on heart morphology or physiology.

To image heart activity, *Drosophila* larvae need to be immobilized. There are a number of techniques which can be used to achieve this [36]. Here wandering stage 3^rd^ instar larvae were immobilized by adhering them, ventral side down, to double-sided sticky tape on a glass slide. This method is simple, fast and easy. However, care must be taken not to overstretch the larvae, as the heart is suspended via alary muscles anchored to the body wall [5], stretching of the body can affect cardiac behavior. Moreover, in time the larvae will dehydrate. Thus all imaging was done directly after larval mounting.

The axial plane at which the heart exhibited its widest diameter was chosen for imaging. The photodetected optical signal was finally converted into images by means of a custom Bitflow SDK based software operating on a Raven BitFlow dual stage frame grabber. Individual stacks of over 100 frames per larva were recorded. After imaging, the system was switched to the stethoscope Doppler OCT regime for audio recording. Immediately after switching, the specimen carrying stage was slightly readjusted till the position where the highest pitch or frequency of the Doppler signals was detected. Then the Doppler signal in that position in each heart was acquired via the audio card of the computer and plotted using Audacity software (http://audacity.sourceforge.net/).

The heart chamber size, the distance between the two sides of heart walls in the heart image frames at the sagittal plane was measured with Image J (http://rsbweb.nih.gov/ij/). Among the recorded total frames in each animal, the frame showing the greatest distance was chosen to represent the end of heart diastolic caliber (EDC). The frame with the least distance was chosen to represent the end of systolic caliber (ESC). Heart shortening fraction was calculated by [(EDC − ESC)/EDC] ×100%. Data recorded at 2 Hz and 5 Hz were pulled and used in this shortening fraction analysis.

The heartbeat tracing in Fig. 3A (bottom panel) also displays the frequency of the Doppler signal produced by the moving wall of the heart chamber. As described in the review by Podoleanu [37], the frequency (f) of the Doppler signal is related to the velocity (v) of movement of the heart wall according to the equation f  = 2 v/λ, where λ is the wavelength of SLD light source (1.3 µm).

### Statistics

Student's t test was used in the statistic analysis.

## Supporting Information

Audio Recording S1 Sound of *Drosophila* heartbeat.
(3.08 MB WAV)

Movie S1 Heart beat in a wild type *Drosophila* larva. The speed of the movie is 5 frames / sec.
(5.23 MB AVI)

Movie S2 The speed of the movie is 5 frames/ sec. Cardiac chamber-shortening contraction in a Tropomyosin mutant larva.
(6.51 MB AVI)

Movie S3 Cardiac pausing in a Tropomyosin mutant larva. The speed of the movie is 5 frames / sec.
(5.09 MB AVI)
